# *South African Family Practice Manual*, fourth edition: Meeting expectations?

**DOI:** 10.4102/safp.v65i1.5820

**Published:** 2023-09-26

**Authors:** Arun Nair, Talat Habib

**Affiliations:** 1 Department of Family Medicine, Faculty of Health Sciences, University of the Free State, Bloemfontein, South Africa; 2 Department of Family Medicine, Robert Mangaliso Sobukwe Hospital, Kimberley, South Africa

## Background

The South African Family Practice Manual (SAFPM), an ongoing project of the South African Academy of Family Physicians (SAAFP), has been in existence since 1995 when its first edition was launched. In 2006, the recognition of family medicine as a speciality in South Africa highlighted the need for comprehensive training resources. In response, further editions of the manual were developed, serving as a reliable training resource for primary health care professionals. This evolving manual covers essential skills such as leadership, management, clinical governance and clinical proficiencies, reflecting the dynamic nature of the speciality.

The manual is a crucial project, fostering the growth of family medicine not only in South Africa but also in other sub-Saharan African countries. By continually adapting to the field’s evolving demands and including essential non-clinical skills, it serves as an indispensable resource for shaping competent healthcare professionals in primary care and family medicine.

## What is new in the fourth edition

The fourth edition of the SAFPM is the most comprehensive to date, spanning over 800 pages and divided into 16 sections. The latest edition underwent revisions in certain sections, such as ‘community-oriented primary care’ and ‘palliative care’, to align with current practices and a new section on ‘rehabilitation skills’ was added to meet evolving professional needs. The manual showcases its forward-looking approach by introducing new skills chapters, encompassing ‘point of care ultrasound’, ‘ketamine anaesthesia’, ‘sexual history’, ‘emergency fasciotomy’ and ‘amputations’. Moreover, it includes a thoughtful teaching and training section, geared towards creating enriching learning environments and elevating training standards, thus nurturing the growth of aspiring family medicine and primary care practitioners.

There was an expansion of the editorial team with the fourth edition to include three new editors: Prof. Hanneke Brits from the University of the Free State, Prof. Mergan Naidoo from the University of KwaZulu-Natal and Prof. Tasleem Ras from the University of Cape Town constituting a more collaborative project involving academics from various departments of family medicine in South Africa. Besides capturing the collective experiences of diverse family physicians, each chapter’s authors also bring specialised expertise in particular skills. Additionally, the book includes contributions from experts in different disciplines, such as dermatology, emergency medicine, paediatrics, public health and plastic surgery.

## Strengths and weaknesses

The new manual aligns seamlessly with the current list of 206 clinical skills essential for training family physicians, making it a vital resource for registrars enrolled in family medicine training programmes, including those preparing for the national fellowship examination as well as postgraduate diploma in family medicine. Additionally, its relevance extends to medical generalists and other primary health care professionals, such as clinical nurse practitioners, clinical associates, clinical trainers and supervisors. The manual covers practical skills relevant to district health care, making it suitable for rural and district health settings, as well as primary care and general practice. At the undergraduate and early career levels, the manual offers a comprehensive, step-by-step guide to honing communication and procedural skills.

Nonetheless, there exist potential areas for improvement in future editions, such as the incorporation of chapters on cross-cultural consultation and patient safety issues with an emphasis on timely reporting of every relevant patient safety incident including near misses. Future editions have the potential to expand and include global themes, such as planetary health, one health, and the integration of artificial intelligence into health systems and research platforms. Emphasising its role as a practical guide, the manual warrants active utilisation and implementation of its content in real-world scenarios. Encouraging regular practice and adherence to standardised procedures, it serves as a dynamic tool rather than a mere reading or reference text. It also focuses on adaptability to evolving evidence and recommendations by avoiding actual clinical management guidelines. Instead, it serves as a practical guide for regular skill practice in diverse settings, with supervisors providing consistent guidance to trainees.

## Final thoughts

From our perspective as clinicians, educators and clinical supervisors, we feel the fourth edition has improved on the previous editions with the added contents and revisions adapting to the evolving training needs and fulfilling the role of the most standardised practical resource in family medicine and primary care. We have used it for bedside teaching and training of various categories of primary care health professionals as well as a reference text for the preparations of formative and summative assessments of students.

We welcome this updated and expanded manual and recommend that every primary care clinician incorporate it into their collection of key clinician references to enhance their practice and skills. The manual’s comprehensive and integrated updates reflect its commitment to staying at the forefront of the field. By providing the latest knowledge and proficiencies, the fourth edition has met the expectations to equip healthcare professionals to excel in the dynamic landscape of family medicine.

