# Erratum: *South African Family Practice Manual, fourth edition*: Meeting expectations?

**DOI:** 10.4102/safp.v65i1.5843

**Published:** 2023-11-22

**Authors:** Arun Nair, Talat Habib

**Affiliations:** 1 Department of Family Medicine, Faculty of Medicine, University of the Free State, Kimberley, South Africa; 2 Robert Mangaliso Sobukwe Hospital, Kimberley, Northern Cape Department of Health, South Africa

In the published book review article, Nair A, Habib T. *South African Family Practice Manual, fourth edition*: Meeting expectations? S Afr Fam Pract. 2023;65(1), a5820. https://doi.org/10.4102/safp.v65i1.5820, an error occurred, being the wrong publisher name was used; Publisher: OUP Southern Africa, Cape Town, in the publication of the book review article. The correct publisher name should have been; Van Schalk Publishers, Cape Town.

The publisher apologises for this error. The correction does not change the book review findings of significance, the overall interpretation of the study’s results, or the scientific conclusions of the book review in any way.

